# Advances in understanding the Th17/Treg balance in systemic lupus erythematosus: implications for treatment and management

**DOI:** 10.3389/fimmu.2026.1795760

**Published:** 2026-04-16

**Authors:** Yi Liu, Ling Xiang, Rui-Juan Cheng, Shu-Yue Pan

**Affiliations:** 1 Department of Rheumatology and Immunology, The Chengdu Wenjiang District People’s Hospital, Chengdu, China; 2 Department of Rheumatology and Immunology, The Chengdu Fifth People’s Hospital, Chengdu, China; 3 Department of Rheumatology and Immunology, West China Hospital, Sichuan University, Chengdu, Sichuan, China

**Keywords:** novel therapies, regulatory T cells, systemic lupus erythematosus, targeted therapies, Th17 cells

## Abstract

Systemic lupus erythematosus (SLE) is a chronic autoimmune disorder characterized by a complex and multifactorial pathogenesis. Recent studies have highlighted the critical role of the disrupted balance between T helper 17 (Th17) cells and regulatory T (Treg) cells in disease initiation and progression. Understanding this imbalance has offered a conceptual framework for developing novel targeted interventions. In this review, we focus on emerging therapeutic strategies aimed at restoring the Th17/Treg balance. We place particular emphasis on the mechanisms and clinical implications of biologic agents, such as IL-17 inhibitors, IL-6 receptor antagonists, low-dose IL-2, and mammalian target of rapamycin (mTOR) pathway inhibitors. Additionally, we explore cell-based therapies, including chimeric antigen receptor T-cell (CAR-T) and CAR-Treg approaches. We also outline ongoing challenges and potential future directions for translating these findings into precise, immune-modulating treatments for patients with SLE.

## 1 Introduction

Systemic lupus erythematosus (SLE) is a chronic, multisystem autoimmune disease characterized by complex pathogenesis and heterogeneous clinical manifestations. It often involves damage to organs and systems such as the kidneys, skin, joints, and lungs. Patients commonly experience common symptoms such as fatigue, arthralgia, erythema, myalgia, and fever (1). The clinical symptoms vary widely among individuals, ranging from mild cutaneous and articular symptoms to severe organ involvement, which may overlap with other diseases and complicate diagnosis and treatment. The heterogeneous nature of SLE makes its diagnosis and treatment challenging, requiring a comprehensive understanding of its diverse presentations and underlying mechanisms. The incidence of SLE in women is approximately nine times higher than in men, with most patients being of childbearing age (2). Studies have shown a higher incidence in high-income countries, with a global incidence of 1.5–11 cases per 100,000 person-years and a prevalence of 13–7,713.5 cases per 100,000 person-years (3). These geographic and demographic differences may be related to environmental factors, genetic background, and socioeconomic conditions.

The pathogenesis of SLE involves the interplay of multiple immune cells and cytokines. Aberrant activation and functional dysregulation of immune cells play a crucial role in which autoantibodies and immune complex formation by B cells are central to disease development (4). These autoantibodies attack self-tissues, leading to inflammation and tissue damage. CD4^+^ T cells not only drive inflammatory responses but also promote B-cell activation and differentiation (5). Among CD4^+^ T-cell subsets, the balance between T helper 17 (Th17) cells and regulatory T (Treg) cells is critical for maintaining immune homeostasis. Accumulating evidence indicates that Th17 overactivation contributes to SLE pathogenesis, whereas impaired Treg function leads to loss of immune tolerance and disease progression (6, 7). Research on the Th17/Treg balance has provided new insights and therapeutic strategies for SLE.

## 2 Mechanistic roles of Th17 and Treg cells in the development of SLE

### 2.1 Th17 cells and SLE

Th17 cells, a subset of CD4^+^ T helper cells identified relatively recently, have drawn substantial attention for their potent proinflammatory activity. These cells secrete a range of cytokines, including IL-17A, IL-17F, IL-21, IL-22, and TNF-α, which collectively recruit inflammatory cells, activate B lymphocytes, and drive the production of autoantibodies (8). Elevated frequencies of Th17 cells and increased IL-17 concentrations have been consistently reported in patients with active SLE compared with those in remission (6, 9). Moreover, increased Th17 and Th1 populations are frequently observed in lupus nephritis, further highlighting their involvement in disease pathogenesis (10).

At the molecular level, the transcription factors signal transducer and activator of transcription 3 (STAT3) and retinoic acid receptor-related orphan receptor γt (RORγt) are indispensable for Th17 differentiation and function (10, 11). Functionally, Th17 cells can be divided into pathogenic and non-pathogenic subsets. Pathogenic Th17 cells are closely linked to autoimmune inflammation, producing abundant proinflammatory cytokines that contribute to tissue injury (12). By contrast, non-pathogenic Th17 cells participate in host defense against extracellular bacteria and fungi, thereby maintaining immune equilibrium (13). Cytokine context dictates the differentiation of these functional subsets. TGF-β, together with IL-6, tends to induce non-pathogenic Th17 polarization, whereas IL-1β, IL-6, and IL-23 promote the generation of pathogenic Th17 cells (14). IL-6 activates the JAK–STAT3 pathway, upregulating RORγt and RORα expression and increasing IL-1R and IL-23R signaling, which stabilizes Th17 lineage commitment (15, 16). IL-23, in turn, drives Granulocyte-Macrophage Colony-Stimulating Factor (GM-CSF) production, a cytokine crucial for Th17 pathogenicity (17). Meanwhile, TGF-β can suppress IL-6-induced SOCS3 expression, thereby prolonging STAT3 activation and sustaining non-pathogenic Th17 activity (18). Collectively, IL-6, IL-23, TGF-β, and RORγt transcriptional regulation serve as core determinants of Th17 differentiation and function, and their dysregulation contributes to aberrant Th17 responses in SLE (16, 19). In addition, the activation of the mammalian target of rapamycin (mTOR) pathway enhances glycolytic metabolism in naïve T cells, thereby facilitating Th17 differentiation and amplifying inflammatory potential (20).

### 2.2 Treg cells and SLE

Treg cells are a specialized subset of CD4^+^ T cells endowed with potent immunosuppressive activity, essential for establishing and maintaining self-tolerance (21). Loss or dysfunction of Treg cells has been directly linked to the pathogenesis of multiple autoimmune disorders, including SLE (20, 22). Experimental studies have demonstrated that the adoptive transfer of Tregs into lupus-prone mice can alleviate inflammation and limit tissue injury, underscoring their therapeutic relevance (23). Treg cells consist of several distinct subtypes—thymus-derived natural Treg (tTreg), peripherally induced Treg (pTreg), and *in vitro*-induced Treg (iTreg)—each contributing uniquely to immune regulation (24). Although the full spectrum of Treg functions in SLE has not been completely delineated, mounting evidence indicates that they exert suppressive effects through several complementary mechanisms, including cytokine secretion, direct cell-to-cell contact, metabolic interference with effector cells, and modulation of antigen-presenting cell (APC) activity (25, 26).

Tregs exert their immunosuppressive effects by secreting a variety of inhibitory cytokines, such as IL-10, IL-35, and TGF-β, which suppress the activity of immune cells (27). In addition, Treg cells can induce programmed cell death in target cells through direct cell-to-cell contact, utilizing molecules such as granzyme and perforin. Tregs also regulate effector function by disrupting cellular metabolism. Cytotoxic T-lymphocyte-associated protein 4 (CTLA-4), a transmembrane receptor critical for Treg stability and suppressive capacity, competes with CD28 on effector T cells for binding to costimulatory molecules CD80 and CD86 on dendritic cells (DCs), thereby attenuating T-cell activation and hindering DC maturation (28). This interaction further promotes the production of indoleamine 2,3-dioxygenase (IDO) and arginase 1, enzymes that deplete tryptophan—an amino acid required for effector T-cell proliferation—resulting in additional immunosuppression (29). Notably, reduced CTLA-4 expression has been documented in patients with SLE and correlates with disease activity (30), suggesting that impaired CTLA-4 signaling contributes to Treg dysfunction. These findings collectively highlight CTLA-4 as a potential therapeutic target in SLE.

Treg cells are phenotypically defined by high expression of CD25 (the IL-2 receptor α chain) and the transcription factor FOXP3, both indispensable for their differentiation and stability (25). FOXP3 serves as a master transcriptional regulator in Treg development, coordinating interactions among various transcription factors and chromatin modifiers. FOXP3 mutations can lead to impaired Treg functionality (31, 32). Growth differentiation factor 7 (GDF7), a member of the TGF-β superfamily, has been shown to enhance Treg function by upregulating FOXP3 and CTLA-4 expression; reduced GDF7 expression in SLE patients may therefore underlie impaired Treg activity (33). Recent studies have also identified alterations in long non-coding RNAs (lncRNAs) that correlate with abnormal Treg phenotypes and SLE disease activity, pointing to an epigenetic layer of Treg regulation (34).

### 2.3 Th17/Treg balance in SLE pathogenesis

**Figure 1:**
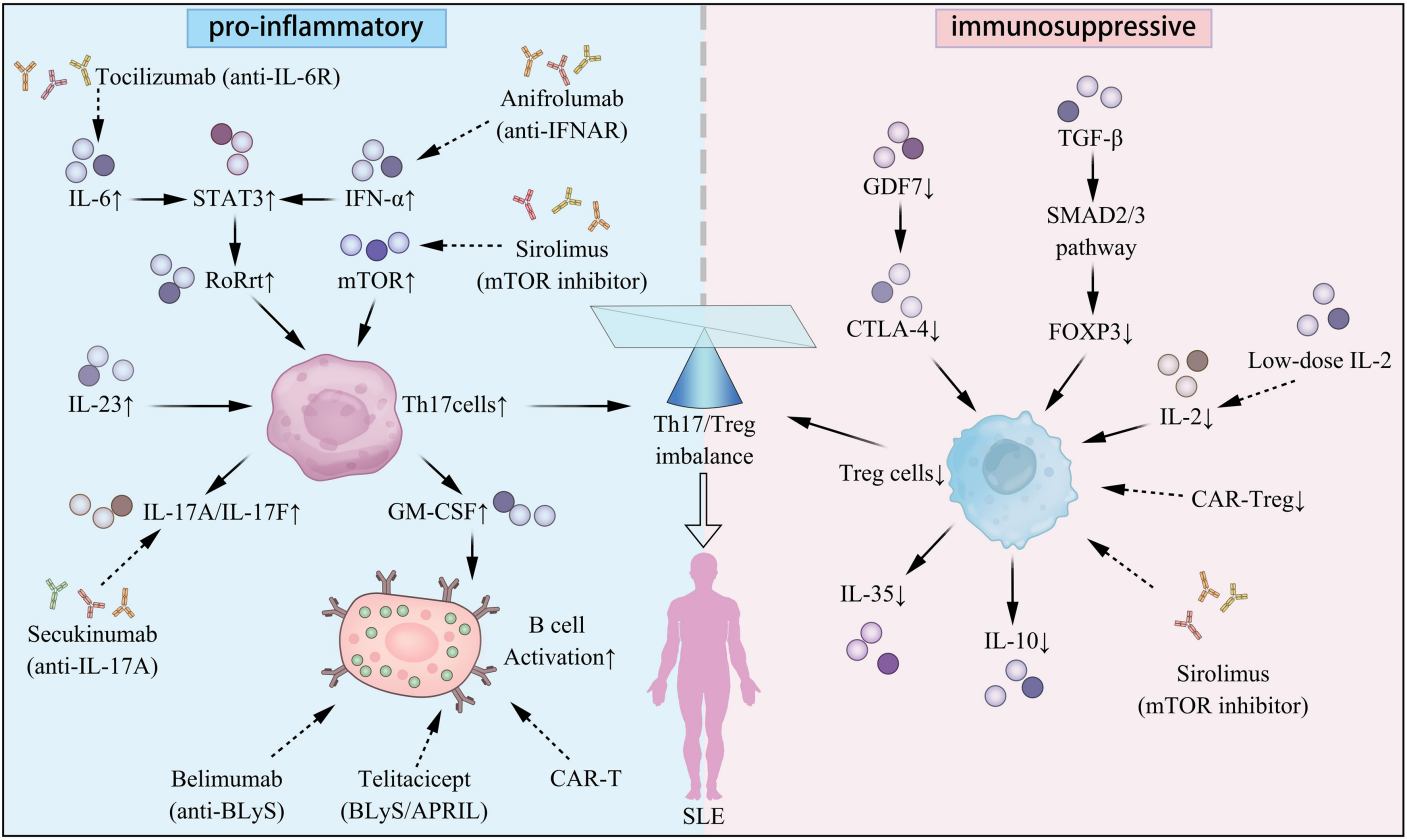
Regulatory mechanisms and therapeutic targets of Th17 cells, regulatory T cells, and their balance in the pathogenesis of SLE. SLE, systemic lupus erythematosus.

As outlined above, Th17 and Treg sustain immune equilibrium through a dynamic interplay that is essential for maintaining self-tolerance and controlled immune activation (**Figure 1**). Mounting evidence suggests that the disruption of this balance is a major immunologic hallmark of SLE and contributes directly to tissue inflammation and organ injury (35, 36).

Although the precise mechanisms underlying Th17/Treg disequilibrium remain to be fully defined, changes in the surrounding cytokine milieu appear to play a central role. In patients with SLE, concentrations of Th17-associated cytokines—including IL-17, IL-23, and IL-6—are markedly increased, generating an inflammatory environment that promotes Th17 differentiation and persistence. In contrast, anti-inflammatory cytokines critical for Treg development and maintenance, such as IL-2 and IL-10, are often reduced. This imbalance weakens Treg suppressive capacity and enhances effector T-cell activation, thereby amplifying systemic inflammation (37). In line with this, Galil et al. (38) reported significantly elevated serum IL-17 and IL-6 levels in individuals with SLE compared with healthy controls, both of which correlated positively with disease activity.

TGF-β acts as a pivotal regulator bridging the Th17 and Treg lineages (39). Acting alone, TGF-β promotes the differentiation of FoxP3^+^ Tregs via SMAD2/3 signaling. However, in the presence of IL-6, TGF-β signaling shifts toward STAT3 activation, inducing RORγt expression and driving Th17 differentiation (14, 40). The elevated IL-6 levels commonly observed in SLE, therefore, skew TGF-β signaling toward Th17 polarization, reinforcing immune imbalance. IL-2 also plays a key role in this process by inducing FoxP3 and suppressing Th17 differentiation, helping to preserve Th17/Treg homeostasis (41). Dysregulated expression of FoxP3 and RORγt further destabilizes this equilibrium and contributes to ongoing inflammation (42).

Type I interferons, particularly IFN-α, further modulate this balance. IFN-α promotes myeloid dendritic cell maturation, enhances autoreactive T-cell activation, and facilitates Th17 differentiation (43). Activated T cells from SLE patients often exhibit increased production of IFN-α and IL-17, thereby sustaining inflammatory cascades (44). Experimental data indicate that loss of IFN receptor signaling specifically in Tregs increases susceptibility to chronic viral infection and tumor growth, implying that IFN pathways may restrain Treg function (45). Viral infections, especially Epstein–Barr virus (EBV), have been implicated as environmental triggers that heighten IFN-α secretion and STAT3 activation, collectively driving Th17 expansion and exacerbating Th17/Treg disequilibrium. In addition, estrogen influences the Th17/Treg axis both directly and indirectly by modulating B-cell responses, which in turn alter T-cell differentiation (46). Collectively, these findings highlight that perturbations in the Th17/Treg axis are central to lupus pathogenesis. Therapeutic strategies aimed at re-establishing this balance may help attenuate inflammation, limit organ damage, and ultimately improve clinical outcomes.

However, when exploring the clinical significance of the Th17/Treg ratio, the impact of methodological heterogeneity must be fully considered. Different studies employ various methods to define and quantify Th17 and Treg subpopulations, including surface markers, intracellular cytokine staining, and transcription factor expression. These technical differences directly affect the comparability of results and their clinical interpretation. First, the phenotypic definition of Treg cells directly influences the interpretation of this ratio. Currently, most studies use “CD4^+^CD25^+^Foxp3^+^” as the identifying marker for Tregs, but this has limitations (6). Research has indicated that the expression of CD25 and Foxp3 is not completely overlapping, and Tregs from different activation stages or tissue sources exhibit differences in suppressive function (47–49). This phenotypic heterogeneity suggests that relying solely on peripheral blood markers may not fully reflect the true functional status of the Treg population. Second, at the transcriptional regulation level, the upregulation of RORγt mRNA expression and the downregulation of Foxp3 mRNA expression in peripheral blood mononuclear cells of SLE patients provide a molecular basis for the Th17/Treg imbalance (50). Notably, recent studies have found that during Th17 differentiation, a stable population of RORγt^+^Foxp3^+^ double-positive cells can emerge (51). These “double-positive T cells” possess plasticity; upon stimulation by inflammatory factors such as IL-6, they can lose their suppressive function, acquire a Th17-like effector phenotype, and secrete IL-17, thereby participating in the pathogenesis of autoimmune diseases (52). This finding suggests that the co-expression status of transcription factors may better reflect the pathological essence of the disease than the simple change in the Th17/Treg ratio, and it also provides a mechanistic basis for explaining why some patients with a “normal” peripheral blood ratio still experience disease progression. Regarding detection methods, multicolor flow cytometry for surface marker and intracellular staining of peripheral blood mononuclear cells is currently the primary method for quantitatively analyzing the aforementioned subpopulations (53). However, the degree of standardization of this method (e.g., stimulation conditions, fixation/permeabilization protocols, and choice of antibody clones) varies among different laboratories, potentially affecting the comparability of results. Furthermore, the limitations of sample sources cannot be ignored. Most existing data originate from peripheral blood tests, but as a systemic autoimmune disease, the key pathological processes of SLE occur in target organs (such as the kidneys). Research indicates that a unique immune microenvironment exists locally in the kidneys, where the cellular composition and functional status differ significantly from those in peripheral blood (54). For instance, tissue-resident memory Th17 cells can persistently produce IL-17 locally in the kidney, driving chronic tissue damage even when systemic inflammation is controlled (55). A 2024 meta-analysis confirmed that the peripheral blood Th17/Treg imbalance is more pronounced in patients with lupus nephritis compared to those without renal involvement (6). Additionally, epigenetic regulation can affect the differentiation of local Tregs in the kidney, thereby altering the Th17/Treg balance (56). Consequently, the peripheral blood ratio may not sensitively reflect the immune microenvironment within target organs, and relying solely on peripheral blood tests may underestimate true disease activity and the risk of tissue damage. Therefore, future research should prioritize the standardization of detection methods and combine multi-site sampling with functional analysis to establish the Th17/Treg ratio as a reliable clinical biomarker in SLE.

## 3 Therapeutic targeting of Th17/Treg balance in SLE

The primary goals of SLE treatment are to control disease activity, delay disease progression, minimize organ damage, and improve patients’ quality of life. Conventional therapies include glucocorticoids, antimalarials, non-steroidal anti-inflammatory drugs (NSAIDs), and immunosuppressants (57). While these drugs can alleviate symptoms and improve survival, their long-term use is associated with serious adverse effects, such as increased infection risk and osteoporosis. Thus, there is an urgent need for safer and more effective therapies. Given that Th17/Treg dysregulation plays a critical role in SLE pathogenesis, therapies aimed at re-establishing this immune balance represent a promising direction for precision treatment. This section focuses on novel biologics targeting specific cytokines or signaling pathways and emerging cellular therapies such as chimeric antigen receptor T-cell (CAR-T) and CAR-Treg, summarizing their mechanisms, current clinical status, and challenges (**Table 1**).

**Table 1:** Emerging Th17/Treg-targeted therapeutic strategies for SLE: mechanisms, safety, and challenges.

Therapy	Target/mechanism	Clinical status and potential	Safety considerations	Major challenges and remarks
Secukinumab	Anti-IL-17A monoclonal antibody; neutralizes Th17 effector cytokine	Approved for other autoimmune diseases; exploratory use in SLE; case reports show efficacy in refractory LN	Infections, neutropenia	Not yet approved for SLE; efficacy and safety require validation in large clinical trials
Tocilizumab	Anti-IL-6 receptor antibody; blocks IL-6 signaling driving Th17 differentiation	Exploratory use in SLE; case reports show efficacy in refractory SLE	Dose-limiting neutropenia, infections	No phase II/III trials; uncertain risk–benefit
Low-dose IL-2	Selectively expands Tregs and restores Th17/Treg balance	Proven safety in SLE and RA; potential first-line therapy for mild to moderate disease	Injection site reactions, flu-like symptoms	Optimal dosing regimen; need for long-term data
Sirolimus	mTOR inhibition; enhances Treg stability, suppresses Th17	Promotes immune rebalancing; synergistic effect with low-dose IL-2; reduces steroid requirement	Hyperlipidemia, cytopenia, mucocutaneous effects	Requires dose optimization and monitoring
Belimumab	Inhibits BLyS activity, reduces autoantibody production	Approved in China; may restore Th17/Treg balance	Infections, infusion reactions	Direct T-cell effects need confirmation
Telitacicept	Dual inhibition of BLyS and APRIL reduces B-cell survival and antibody production	Approved in China; significantly improves disease activity	Infections, injection site reactions	No direct evidence yet of effects on Th17/Treg balance
Anifrolumab	Anti-IFNAR antibody blocks type I interferon signaling	Effective in moderate to severe SLE; reduces steroid use	Infections, infusion-related reactions	Direct effects on T-cell subsets need further elucidation
CAR-T therapy	Engineered T cells targeting pathogenic B cells (e.g., CD19^+^) to deplete autoantibody sources	Sustained drug-free remission in refractory SLE; B-cell aplasia with recovery	CRS, ICANS, prolonged cytopenia, hypogammaglobulinemia	Very limited evidence; patient selection critical; long-term safety unknown
CAR-Treg therapy	Engineered Tregs with enhanced antigen-specific suppressive activity to restore immune tolerance	Preclinical development; offers high specificity and reduced systemic immunosuppression	Theoretical lower systemic toxicity	Technical and regulatory hurdles remain; clinical evidence still limited

BLyS, B-lymphocyte stimulator; APRIL, a proliferation-inducing ligand; CRS, cytokine release syndrome; ICANS, immune effector cell-associated neurotoxicity syndrome; IFNAR, type I interferon receptor; LN, lupus nephritis; mTOR, mammalian target of rapamycin; RA, rheumatoid arthritis.

### 3.1 Biologic therapies

Recent advances in biologics have opened new possibilities for SLE treatment. IL-17A, a hallmark cytokine of Th17 cells, plays a pivotal role in initiating and propagating autoimmune responses (58). Secukinumab, a monoclonal antibody targeting IL-17A, is approved for psoriasis, psoriatic arthritis, and ankylosing spondylitis (59). Its use in SLE remains investigational, with evidence currently limited to preclinical studies and case reports. A case study demonstrated that secukinumab successfully alleviated symptoms in a patient with refractory lupus nephritis complicated by psoriasis (60). Another case report of refractory lupus nephritis similarly demonstrated a significant reduction in proteinuria and sustained remission for over 24 weeks after 12 weeks of secukinumab treatment (61). While these observations are encouraging, large-scale clinical studies are still required to further validate its efficacy and safety in patients with systemic lupus erythematosus.

In addition, IL-6 is a pivotal cytokine in the pathogenesis of SLE that stabilizes Th17-cell differentiation. Tocilizumab is a humanized monoclonal antibody targeting the IL-6 receptor. A phase I open-label study involving 16 patients with moderate to active SLE demonstrated clinical improvement in 53% of subjects, alongside reductions in anti-double-stranded DNA antibodies and circulating plasma cell levels (62). However, dose-limiting neutropenia and infection events were observed, and no phase II/III trials have been completed to date. Subsequent research on tocilizumab in SLE has been largely confined to case reports and small case series. For instance, case reports indicate that tocilizumab achieved clinical remission in three refractory SLE cases presenting with persistent fever, arthritis, serositis, and hemolytic anemia (63, 64). Consequently, the role of IL-6 blockade in SLE remains unclear, necessitating further studies to define its risk–benefit profile.

Other immunomodulatory strategies show promise. Currently, low-dose IL-2 has been proven to have good safety in autoimmune diseases such as rheumatoid arthritis (RA) and SLE (65, 66). The meta-analysis by Su et al. showed that after low-dose IL-2 treatment, patients’ Tregs were significantly increased, and the Th17/Treg imbalance was corrected (67). The first international multicenter randomized double-blind placebo-controlled phase II trial (LUPIL-2) conducted by Humrich et al. further confirmed that low-dose IL-2 therapy can increase Treg frequency, reduce disease activity scores, and allow for glucocorticoid tapering (68). The most common side effects were injection site reactions and transient flu-like symptoms (69). For patients with severe autoimmune diseases, IL-2 may be combined with other anti-inflammatory therapies (70). Current research is dedicated to optimizing treatment regimens through larger trials and exploring biomarkers that can predict efficacy, aiming to achieve more precise personalized treatment.

Sirolimus, a macrolide immunosuppressant that inhibits mTOR activity, has been shown to enhance the metabolic fitness and functional stability of Treg cells while suppressing Th17 differentiation and IL-17 secretion, thereby promoting the rebalancing of the Th17/Treg axis (20). A phase I/II trial of sirolimus in patients with SLE reported significant reductions in disease activity scores and steroid use over 12 months (71). A retrospective analysis by Ding et al. (72) similarly reported that add-on sirolimus improved clinical manifestations, serologic abnormalities, and disease activity in individuals with SLE. Furthermore, a study by Bai et al. suggested that sirolimus may also hold therapeutic potential for the treatment of lupus nephritis (LN) (73). Notably, combining low-dose IL-2 with rapamycin produces synergistic effects, significantly increasing Treg numbers, reducing disease activity, and lowering glucocorticoid use (74). However, sirolimus is associated with certain risks, including hyperlipidemia, cytopenia, and mucocutaneous reactions, necessitating rigorous therapeutic drug monitoring. Further high-quality studies are warranted to confirm both the efficacy and safety of sirolimus.

In China, approved biologics targeting B cells include telitacicept and belimumab. Telitacicept is a dual-target biologic that inhibits both B-lymphocyte stimulator (BLyS) and a proliferation-inducing ligand (APRIL), thereby reducing B-cell survival, differentiation, and autoantibody production, significantly improving disease activity (75). A phase IIb clinical trial confirmed its efficacy and acceptable safety in SLE (76). However, direct evidence for effects on the Th17/Treg balance remains lacking. Belimumab, a fully human monoclonal antibody targeting BLyS, has been shown to reduce disease activity in East Asian SLE patients while allowing glucocorticoid tapering (77). Preliminary findings suggest that belimumab may help restore the Th17/Treg balance (78), although the evidence is indirect and requires confirmation.

Type I interferons, particularly IFN-α, play a major role in SLE pathogenesis through the activation of the JAK–STAT pathway, which promotes Th17 differentiation and suppresses Treg function (79). Anifrolumab, a monoclonal antibody targeting the type I interferon receptor (IFNAR), blocks this signaling (80). Clinical studies have shown that anifrolumab effectively controls disease activity in moderate to severe SLE while reducing glucocorticoid use (81). A phase III trial by Morand et al. (82) further supports its efficacy, and comparative analyses suggest that anifrolumab may offer superior clinical benefit compared to belimumab (83). Common adverse reactions of anifrolumab include infections and infusion-related reactions (84). It has been approved in the United States, Europe, and several other regions for moderate to severe SLE.

### 3.2 Cellular therapies

The rapid development of immune cell therapies has opened new avenues for treating autoimmune diseases. CAR-T therapy, which has achieved remarkable clinical success in hematologic malignancies, is now being explored for autoimmune diseases, including SLE (85).

The rationale derives from the central role of B-cell hyperactivity and autoantibody production in SLE pathogenesis (86). Conventional B-cell depletion therapies (e.g., rituximab) have limitations, including incomplete depletion, repeated dosing requirements, and adverse effects. By contrast, CAR-T therapy involves engineering patients’ own T cells to express chimeric receptors targeting B-cell antigens such as CD19, thereby specifically eliminating autoreactive B cells and indirectly reducing Th17 activation. In a landmark study, five patients with refractory SLE achieved sustained disease remission after CAR-T treatment, accompanied by marked reductions in autoantibody levels and no severe toxicities (87). CAR-T therapy also facilitates immune tolerance reconstitution during B-cell reconstitution (88). Mougiakakos et al. (89) also reported a case of successful CAR-T cell therapy in a patient with relapsed/refractory systemic lupus erythematosus. Although CD19 CAR-T therapy has achieved breakthrough results, its limited ability to eliminate long-lived plasma cells (LLPCs) may contribute to disease relapse. In a phase 1 trial (90), Hong et al. developed ICG318, a dual-target BCMA-CD19 CAR-T designed to eliminate both B cells and LLPCs. Of these, 83% achieved stringent complete remission (sCR) with favorable tolerability, providing preliminary evidence for dual-target CAR-T therapy in SLE.

Despite these promising results, CAR-T therapy faces multiple challenges. First, safety concerns primarily include cytokine release syndrome (CRS), immune effector cell-associated neurotoxicity syndrome (ICANS), and on-target off-disease toxicity (91). Second, the complex manufacturing process and high costs limit widespread accessibility. Additionally, critical issues remain to be elucidated, including the long-term efficacy of CAR-T cells, optimal lymphodepletion regimens, risk of malignant transformation, and effects on fertility. Current studies are limited by small sample sizes and the absence of standardized treatment protocols. Future research should focus on large-scale randomized controlled trials to further validate the long-term efficacy and safety of CAR-T therapy. Concurrently, the exploration of novel technological approaches, including dual-targeting, universal CAR-T, and *in vivo* CAR-T, is essential to enhance treatment accessibility and precision.

These research data preliminarily indicate that CAR-T cell therapy may achieve rapid remission in refractory SLE. Although these findings highlight its therapeutic potential, CAR-T therapy for SLE remains in early clinical development, and further trials are required to evaluate long-term efficacy and safety.

Another promising approach is CAR-Treg therapy, which involves engineering Tregs with chimeric receptors to enhance their antigen-specific suppressive function, restoring immune balance and reducing autoimmune responses (92). In the first reported case of autologous Treg adoptive therapy in cutaneous lupus, infusion of *ex vivo* expanded CD4^+^ Tregs increased *in vivo* Treg activity and reduced inflammation (93). Compared with conventional therapies, CAR-Treg offers greater precision in immune modulation, potentially avoiding systemic immunosuppression. However, this strategy remains in early exploratory phases, with substantial technical and regulatory hurdles, including Treg stability, antigen specificity, and manufacturing challenges. Clinical evidence remains extremely limited.

## 4 Conclusion

The Th17/Treg axis plays a central role in SLE pathogenesis. Overactivation of Th17 cells and their effector cytokines drives inflammation and tissue damage, whereas Treg cells maintain immune tolerance and homeostasis. The disruption of this balance, regulated by key cytokines such as IL-6, TGF-β, IL-2, and type I interferons, as well as signaling pathways including JAK–STAT and mTOR, contributes to disease progression.

Conventional therapies, including glucocorticoids, antimalarials, NSAIDs, and immunosuppressants, remain the mainstay of treatment but are limited by adverse effects and lack of specificity. In recent years, targeted biologics and cellular therapies have emerged as promising alternatives. Biologics targeting IL-17A, IL-6R, BLyS/APRIL, or IFNAR, as well as low-dose IL-2 and mTOR inhibitors, have demonstrated potential to rebalance Th17/Treg immunity and improve clinical outcomes, although evidence levels vary considerably. Low-dose IL-2 and anifrolumab have the strongest supportive data from controlled trials, while evidence for IL-17 and IL-6 blockade remains preliminary. Novel cellular therapies such as CAR-T and CAR-Treg offer unprecedented opportunities for immune tolerance reconstitution in refractory disease, but currently represent experimental approaches with substantial risks and uncertainties.

The precise mechanisms regulating the Th17/Treg balance in SLE are not fully understood. The efficacy and safety of emerging therapies need to be verified in large-scale, well-controlled clinical trials with long-term follow-up. At the same time, the identification of biomarkers that predict treatment response is crucial for personalized treatment selection.

Despite these challenges and continued advances in immunology and therapeutic technology, Th17/Treg-targeted precision immunotherapy may become a cornerstone of SLE treatment, particularly for refractory disease, ultimately improving long-term outcomes and quality of life for patients.
